# A Simple and Sensitive HPLC Method for Fluorescence Quantitation of Doxorubicin in Micro-volume Plasma: Applications to Pharmacokinetic Studies in Rats

**Published:** 2015

**Authors:** Marjan Daeihamed, Azadeh Haeri, Simin Dadashzadeh

**Affiliations:** a *Department of Pharmaceutics, School of Pharmacy, Shahid Beheshti University of Medical Sciences, Tehran, Iran. *; b *Pharmaceutical Sciences Research Center, Shahid Beheshti University of Medical Sciences, Tehran, Iran.*

**Keywords:** Doxorubicin, HPLC, Fluorescence, Pharmacokinetics, Rat plasma, Small sample volume

## Abstract

A validated HPLC method was developed to determine the doxorubicin concentration in a small volume of rat plasma (60 µL) with convenient fluorescence detection. Sample preparation includes a simple one-step liquid-liquid extraction using a minimum amount of organic solvent, with extraction recovery more than 95%. The analysis was accomplished using PerfectSil C18 column maintained at 35 °C and a mobile phase consisted of acetonitrile and water (32:68, v/v; pH=2.6). The flow-rate was kept at 1 mL/min and the column effluent was monitored with a fluorescence detector at an excitation and emission wavelength of 470 and 555 nm, respectively. The detection limit was 5 ng/mL. No analytical interference was observed from endogenous components in the rat plasma. This method was feasibly applied to the pharmacokinetic study of 5 mg/Kg of doxorubicin after the intravenous administration to rats.

## Introduction

Doxorubicin hydrochloride (Dox) is a broad spectrum antineoplastic drug widely used in the management of different cancers including hematologic malignancies and solid tumors (1, 2). However, in spite of its great efficacy, undesirable side effects such as dose-related cardiotoxicity, myelosuppression and development of drug resistance have impeded its clinical utility (3, 4). In attempts to overcome these problems, several drug delivery approaches including nanoparticles, liposomes, polymeric micelles and polymer conjugates, with passive and active targeting mechanisms, have been developed to attenuate the toxicity and improve the overall therapeutic efficacy of doxorubicin (5-8).

One of the major steps in the optimization of a carrier-based drug delivery system is assessment of the fate of drug through pharmacokinetic analysis of diverse formulations in laboratory animals (9). Rats and mice are most widely used species for routine pharmacokinetic studies due to their cost effectiveness and ease of handling in both dosing and blood sampling processes (10, 11).

Discrete blood sampling is a common method used in pharmacokinetic studies performed in small rodents, especially in mice. In addition to the consumption of a large number of animals, the possibility of inter-animal variability and dosing error brings limitations to discrete sampling method (12). On the other hand, taking large volumes of blood in the serial blood sampling would perturb haemodynamic physiology of animals and results in unreliable pharmacokinetic data (13). Therefore, pharmacokinetic studies conducting serial blood sampling using small volumes of plasma are desirable. This is the case for a drug like doxorubicin that is widely used for drug delivery applications.

Various analytical methods including HPLC with UV (14-16), fluorescence (17-21), tandem mass spectrometry (22, 23), electrochemical (24) and chemiluminescence (25) detection have been published for the analysis of doxorubicin in plasma. However, most reported methods are mainly designed for human biological samples and require large sample volumes (18, 24, 26). Only a few of these analytical methods use small sample volume and are appropriate for pharmacokinetic studies in laboratory animals such as rats and mice (20-23). 

As mentioned earlier, considering the total blood volume that can be removed without significant disturbance to the animal’s normal physiology (27), an ideal analytical method requiring very small sample volume is favored to permit the collection of pharmacokinetic data from single animal experiments.

To the best of our knowledge, most of HPLC methods designed for pharmacokinetic studies of doxorubicin in laboratory animals require at least 100-200 µL of plasma (27). Some of these methods use MS detection (22, 23) that needs special requirements and is not affordable for most laboratories. Other methods that utilize UV or fluorescence detectors suffer from the low sensitivity (28, 29) or long analytical run times (17). On the other hand, sample preparation methods in these studies include protein precipitation which would increase the analytical column pressure (17, 28, 30) , solid phase extraction (31, 32) that is expensive and labor-intensive or liquid- liquid extraction with high amounts of organic solvent (33-35). 

In 2009, Kuroda *et al*. (25) introduced an HPLC method based on photosensitization reaction followed by peroxyoxalate chemiluminescence detection to analyze doxorubicin in 50 µL of rat plasma. Although this method is very sensitive, chemiluminescence detector is not available in a typical laboratory.

The method proposed by Balthasar *et al*. (19) is the only sensitive HPLC method reported for the determination of doxorubicin in a very small plasma volume (20 µL) using convenient fluorescence detection. However, adding Perchloric acid (35%, v/v) to plasma is required for the protein precipitation that can deteriorate the analytical column and harm the injection port in long term use, due to either high acidity or inadequate precipitation of the protein contents in the samples. Injection of the acid supernatant also leads to numerous late eluting peaks which are time consuming and gradient elution is needed to remove them (36, 37). 

In this paper, we report a fully validated, isocratic HPLC method, for sensitive doxorubicin quantification in small volumes of plasma (60 µL) using a simple one-step liquid-liquid extraction with minimum organic solvent consumption. The method employs a convenient fluorescence detector and enables the quantification of doxorubicin in a large number of plasma samples during a reasonable run time (10 min).

Our method has been successfully applied to determine the pharmacokinetics of doxorubicin after IV administration in rats.

## Experimental

*Reagents and chemicals*

Doxorubicin hydrochloride and daunorubicin hydrochloride were purchased from Sigma Chemical Company (St. Louis, MO, USA). Analytical grade chloroform, HPLC grade acetonitrile, methanol, ethyl acetate and methyl tert-butyl ether (MTBE) were purchased from Merck (Darmstadt, Germany). Ultrapure water was obtained using a Millipore Direct-QTM (Millipore Corporation, Bedford, MA, USA).

*Instrumentation*

The HPLC system consisted of a Wellchrom K-1001 HPLC pump, a Wellchrom online degasser and a Rheodyne auto injector equipped with a 50 µL loop were all from Knauer coupled with a fluorescence detector (RF-10A XL Shimadzu). The chromatographic data was acquired by Chromgate 3.1 software from Knauer. 

*Chromatographic conditions*

The chromatographic separation was performed on a PerfectSil C18 column (4.6 × 150 mm, 5 μm particle size, MZ-Analysentechnik, Mainz, Germany). The optimum mobile phase consisting of acetonitrile and water (32: 68, v/v), pH adjusted to 2.6 with 85% orthophosphoric acid was delivered isocratically at a flow rate of 1 mL/min. The column temperature was maintained at 35 °C and excitation and emission wavlengths were set at 475 and 555 nm, respectively. The injection volume was 50 µL.

*Preparation of stock solutions and standards*

Stock solutions (1 mg/mL) of doxorubicin and daunorubicin (IS) were separately prepared in methanol and stored at -20 °C.

To prepare working solutions of doxorubicin, the stock solution was diluted with water to give concentrations of 1, 5, 10, 50, 100, 200, 500 µg/mL. Solutions containing 800 ng/mL IS were also prepared by serial dilutions of stock solution with methanol. 

Calibration standards of doxorubicin were freshly prepared by spiking appropriate amounts of working solutions in pooled drug free plasma at concentrations of 5, 10, 20, 50, 70, 100, 200, 500, 700 and 1000 ng/mL. Quality control (QC) samples in the rat plasma were prepared at concentrations of 10, 50, 100, 500 and 1000 ng/mL. The spiked samples were then treated following the sample preparation procedure as indicated below.

*Sample preparation*

To 60 µL of plasma sample, 50 µL of IS solution (800 ng/mL daunorubicin hydrochloride in methanol) was added and vortex-mixed for 30 s. The extraction of drug was performed by adding 900 µL of a mixture of chloroform/methanol (4:1, v/v). After vortex mixing for 10 min and centrifugation (10 min, 10000 g), the organic phase was collected, transferred to a clean tube and evaporated to dryness under a stream of nitrogen at 40 °C. Dry residues from plasma were dissolved in 60 µL of mobile phase, and after centrifugation for 5 min (10000 g), 50 µL of the supernatant was injected into the chromatographic column.

*Method validation*

The developed method was validated in terms of selectivity, linearity, accuracy and precision (intra and inter-day variability), limits of detection (LOD) and quantification (LOQ), recovery and stability under different storage conditions.

*Selectivity*

In order to verify the selectivity of the method, blank plasma samples of six different rats were analyzed and possible interferences with doxorubicin and IS were checked by visual comparing of chromatograms.

*Linearity*

To evaluate the linearity of HPLC method, plasma calibration curves of at least 9 points were constructed using freshly prepared spiked samples, by plotting the peak-area ratio of doxorubicin to internal standard versus the nominal concentration of doxorubicin. Linearity of the method was established by the least-squares linear regression analysis.

*Precision and accuracy*

The precision of the method is expressed by the relative standard deviation (RSD %) of replicate measurements as a measure of random error. Accuracy of analytical method is reported as a relative error (RE %) and defines the difference between measured and nominal concentrations.

The intra-day and inter-day precisions were determined by analyzing five replicates of QC samples on the same day and three times on three days, respectively.

*Extraction recovery*

The extraction recovery of doxorubicin was determined at three concentrations of 10, 100 and 500 ng/mL by comparing the responses obtained from processed plasma samples with those obtained from aqueous standard solutions (n=5). The extraction recovery of the IS was calculated at the concentration of 800 ng/mL as well.

*Limit of detection (LOD) and Limit of quantitation (LOQ)*

Limit of detection (LOD), defined as the lowest detectable concentration is considered a concentration that has a signal to noise ratio of 3:1 (38). The limit of quantification (LOQ) was taken as the lowest concentration that can be accurately (relative error < 20%) and precisely (RSD < 20%) determined (39).

*Stability*

The stability of QC samples was established under following conditions: after going through three freeze-and-thaw cycles, from -20 °C to room temperature (freeze-thaw stability), short term stability at room temperature for the period of routine sample work-up (at least 3 h), stability of dry extract and long term stability in plasma and stock solutions at storage conditions (-20 °C) for 1 month.

*Pharmacokinetic study*

*Animal treatment*

Male Sprague-Dawley rats weighing 200–220 g were purchased from the Razi Institute of Iran (Tehran, Iran). Each rat was housed in a cage with a 12 h light/12 h dark cycle at ambient temperature (21-22 °C) and the relative humidity of 55 ± 5%. The rats were fasted overnight before experimentation and had ad libidum access to water. All protocols and procedures were approved by the local ethics committee for animal experiments of Shahid Beheshti University in Tehran, Iran.

A solution of doxorubicin (1 mg/mL in normal saline) at a dose of 5 mg/Kg was administered intravenously (I.V.) via the rat tail vein and blood samples (150 µL) were taken from the tail vein before (blank sample) and after the drug administration at several time points (5 min, 0.25, 0.5, 1, 2, 4, 6, 8, 10, 12, 24 and 48 h) in tubes containing EDTA as an anticoagulant. Each blood sample was gently inverted several times to ensure complete mixing with the anticoagulant. After centrifugation at 5000 g for 10 min, plasma was separated and stored at -20 °C until analysis.

*Pharmacokinetic analysis*

Concentrations of doxorubicin in plasma samples were analyzed by the developed HPLC method. Plasma concentration versus time data were analyzed and pharmacokinetic parameters including elimination rate constant (K), elimination half-life (t_1/2_), mean residence time (MRT), volume of distribution at steady- state (Vss), systemic clearance (Cl) and the area under the plasma concentration versus time curve (AUC_0-__∞_) in plasma were determined by a noncompartmental analysis (40). 

The elimination rate constant (K) was estimated by the least-square regression of plasma concentration-time data points in the terminal log-linear region of the curves. The Half-life was calculated as 0.693 which was divided by K. AUC_0-__∞_ was calculated using the trapezoidal rule with extrapolation to infinity. The clearance was calculated by dividing the dose by AUC_0-__∞_. The volume of distribution at steady- state (Vss) and MRT was calculated using the following non-compartmental equations:

MRT = AUMC / AUC_0-__∞_

Vss = Cl / MRT

In previous equations, AUMC (area under the first moment curve) is the area under the curve of C × t versus t from time zero to infinity.

## Results and Discussion

Until today, numerous methods have been published for the analysis of doxorubicin in plasma. However, methods for analyzing very small plasma samples (<100 µL) that are favored during animal pharmacokinetic studies are somehow limited (25) , (19). We designed an HPLC method for analysis of doxorubicin in 60 µL of rat plasma that is utilizable in a typical laboratory for a large number of samples. Validation criteria have been met and the method has adequate characteristics to assure reliable results.

*Selection of chromatographic conditions*

A variety of chromatographic conditions and sample preparation methods were studied to achieve optimum conditions for reasonable separation of analyte peak from other interfering peaks related to plasma endogenous components.

Using a PerfectSil C18 column (4.6 × 150 mm, 5 μm particle size), a variety of mobile phases comprising several combinations of aqueous (0.01 M potassium dihydrogen phosphate buffer or deionized water) and organic solvents (methanol or acetonitrile) were tested to provide sufficient resolution between doxorubicin, IS and plasma interfering peaks. We tried not to use buffers in the composition of mobile phase, as long-term usage of buffers may harm the chromatographic column. The presence of methanol as the main organic eluent in the mobile phase composition resulted in a long run time, so a mixture of water and acetonitrile was used. The best results were obtained with the mobile phase consisted of acetonitrile: water (32:68, v/v) and a pH of 2.6 adjusted with orthophosphoric acid. Increasing the column temperature to 35 °C improved the peak shapes of doxorubicin and IS, and decreased the total run time to 10 min without any need for a post-run wash.

*Sample preparation*

As a simple and rapid method, Protein precipitation has been used extensively for the preparation of doxorubicin containing plasma samples for HPLC analysis (17, 28, 30), but increasing of column pressure has been reported as a common problem in the long term use of these methods. Articles using solid phase extraction (31, 32) have also been published for the doxorubicin analysis in plasma. However, a simpler and rather cost effective method for the analysis of doxorubicin is preferred.

A one step liquid-liquid extraction method with minimum amount of different solvents (900 µL) including acetonitrile, methanol, ethyl acetate, chloroform, MTBE and a mixture of chloroform/methanol (4:1, v/v) for extraction of doxorubicin from 60 µL of rat plasma was tested.

Recovery values of doxorubicin from rats employing the above mentioned solvents have been shown in the Table 1. Using the mixture of chloroform/methanol (4:1, v/v) as the extraction solvent resulted in an acceptable chromatogram and recovery values more than 95%. Therefore, it was selected as the suitable extraction solvent.

**Table 1:** Doxorubicin recoveries following extraction from rat plasma with different solvents (n=3).

	**% Recovery**		
**Extraction solvent**	**Doxorubicin** **10 ng/mL**	**Doxorubicin** **100 ng/mL**	**Internal standard** **100 ng/mL**
Acetonitrile	74.29 ± 5.41	76.47± 3.22	73.42 ± 3.84
Methanol	57.88 ± 4.11	65.33 ± 3.17	69.26 ± 3.22
Ethyl acetate	47.29 ± 3.96	56.47 ± 2.21	70.23 ± 4.32
Methyl tert-butyl ether (MTBE)	39.45 ± 2.17	42.71 ± 1.58	51.38 ± 2.67
Chloroform	82.52 ± 5.09	84.12 ± 3.72	79.7 ± 4.20
Chloroform: Methanol (4:1)	97.42 ± 2.22	98.57 ± 2.48	97.21 ± 1.74

*Method validation*

*Selectivity*

**Figure 1:**
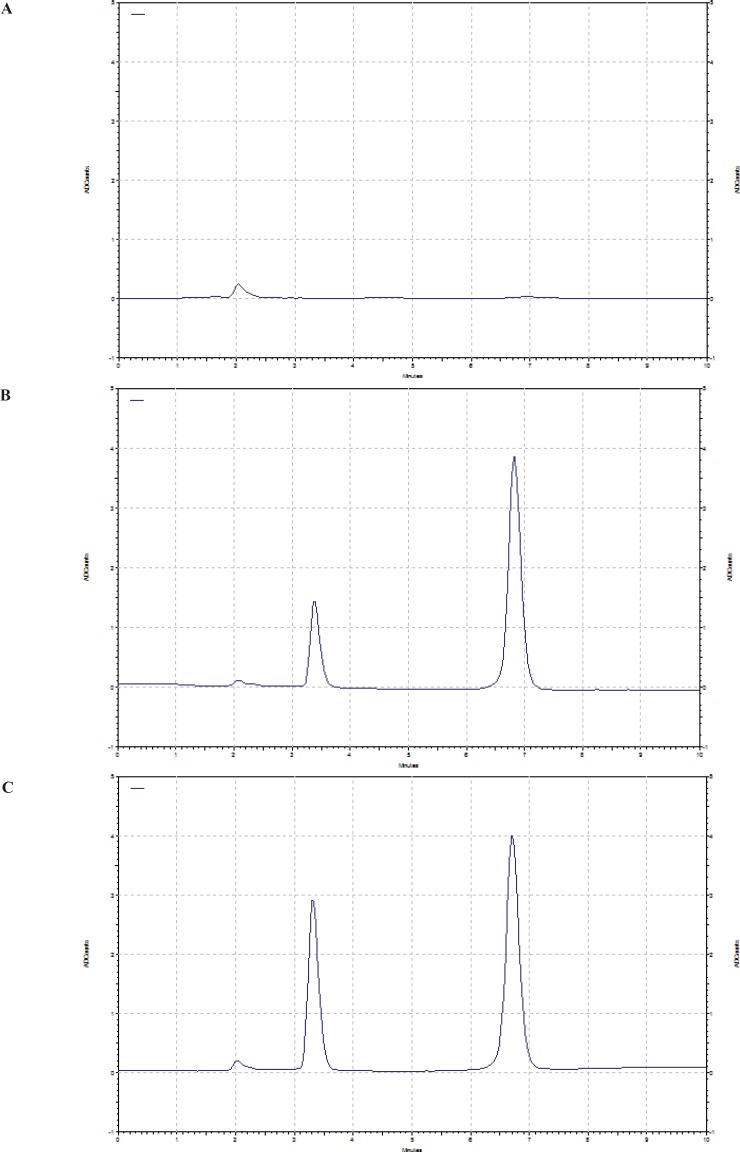
Chromatograms of A) blank rat plasma, B) plasma spiked with 100 ng/mL doxorubicin and C) plasma sample 4 h after I.V. administration of 5 mg/Kg of doxorubicin to rat.

Comparing chromatograms of six different sources of the blank rat plasma showed no interfering peaks in the eluting positions of doxorubicin and IS. Figure 1 shows the typical chromatogram for blank plasma, plasma spiked with 100 ng/mL doxorubicin and plasma 4 h after I.V. administration of 5 mg/Kg doxorubicin solution to the rat. Retention times of doxorubicin and IS were about 3.5 and 6.8 min, respectively.

*Linearity*

A good linearity was observed over the concentration ranges of 5–1000 ng/mL between peak-area ratio of doxorubicin to internal standard and the respective doxorubicin concentrations in plasma. The regression equation was y= 0.0015 x– 0.0087 with an excellent correlation coefficient (r^2 ^> 0.9997).

*Precision and accuracy*

The results concerning the accuracy and intra and inter-day precision of the method have been shown in the Table 2 for each QC level of doxorubicin. Relative standard deviation and relative error values higher than 15% were not accepted except at LOQ, where it should not deviate by more than 20%.

The intra and inter-day precision were less than 5.14 and 9.64%, respectively, and the relative error was between 0.72 - 11.41% which indicated the acceptable accuracy and precision of the developed method.

**Table 2:** The Intra-day and Inter-day precision and accuracy of analytes (n=5).

**Nominal concentration (ng/mL)**	**Measured concentration (ng/mL)**	**SD** **(ng/mL)**	**RSD%**	**Relative error%**
Intraday assay				
10	8.86	0.45	5.14	11.41
50	46.18	1.62	3.55	7.64
100	95.76	1.82	1.97	4.24
500	488.60	11.73	2.41	2.28
1000	992.80	18.57	1.87	0.72
Inter-day assay				
10	9.06	0.64	7.11	9.42
50	47.44	4.62	9.64	5.13
100	96.09	3.30	3.43	3.91
500	490.55	14.42	2.94	1.89
1000	984.80	35.45	3.63	1.52

*Extraction recovery*

The percentage of recovery for doxorubicin was 95.1 ± 5.1, 98.8 ± 3.8, 99.0 ± 2.6, at concentrations of 10, 100 and 500 ng/mL respectively and the recovery of IS was 95.2 ± 3.5 at 800 ng/mL.

*Limit of detection (LOD) and Limit of quantitation (LOQ)*

The limit of detection of doxorubicin was determined as the concentration of drug corresponding to a signal-to-noise ratio of 3:1 in plasma and obtained about 2 ng/mL. The limit of quantification was 5 ng/mL. The method shows a high sensitivity compared to some other methods.

*Stability*

To ensure the reliability of results in relation to handling and storing plasma samples, stability studies were carried out. In each situation, deviations more than ± 5% from the initial concentration were considered unstable. As shown in Tables 3 and 4, doxorubicin was found to be stable in the rat plasma for 3 h at ambient temperature, after three freeze–thaw cycles and storage for 1 month at -20 °C. The obtained results revealed that any spontaneous degradation did not occur during the sample routine analysis and storage. Dried plasma extracts of doxorubicin were found to be stable for 1 month at -20 °C. According to the stability results, in our study collected samples were not stored at ambient temperature for more than 3 h and analyses of all samples were finished before storage for 1 month. 

**Table 3:** Stability of doxorubicin in rat plasma after freeze -Thaw cycles (n=3).

**Number of cycles**	**%** **Remained**		
	**10 ng/mL**	**100 ng/mL**	**500 ng/mL**
First cycle	98.13 ± 1.19	98.52 ± 0.98	99.15 ± 0.65
Second cycle	96.62 ± 1.31	97.44 ± 0.79	97.33 ± 0.73
Third cycle	95.96 ± 0.73	96.32 ± 1.20	96.71 ± 0.97

**Table 4:** Stability of doxorubicin in rat plasma at ambient temperature (n = 3).

**Time (h)**	**%Remained**		
	**10 ng/mL**	**100 ng/mL**	**500 ng/mL**
0.5	98.77 ± 1.03	98.44 ± 0.48	99.21 ± 0.85
1	98.60 ± 0.62	97.83 ± 0.53	98.83 ± 0.29
2	97.93 ± 0.81	97.40 ± 0.51	99.03 ± 0.68
3	97.18 ± 0.41	97.35 ± 0.72	98.56 ± 0.53

*Application of method to pharmacokinetic study*

In order to test the applicability of the method to pharmacokinetic studies of doxorubicin, the proposed HPLC method was used for determination of the drug concentrations in the rat plasma after a single-dose administration of doxorubicin. Using a small volume of plasma in our method enables a multiple blood sampling from each animal. Samples at concentrations over the calibration ranges were determined by the 10-fold dilution.

**Table 5:** Pharmacokinetic parameters of doxorubicin after I.V. administration to rats (n=6).

Dose, mg/Kg	5.00
AUC_0-__∞ _(µg h mL ^-1^)	9.01 ± 1.91
K _el _(h ^-1^)	0.09 ± 0.01
t_1/2 _(h)	7.88 ± 1.01
MRT (h)	5.92 ± 0.92
Cl (mL Kg h ^-1^)	554.94 ± 10.74
Vss (mL Kg ^-1^)	93.74 ± 8.32

**Figure 2:**
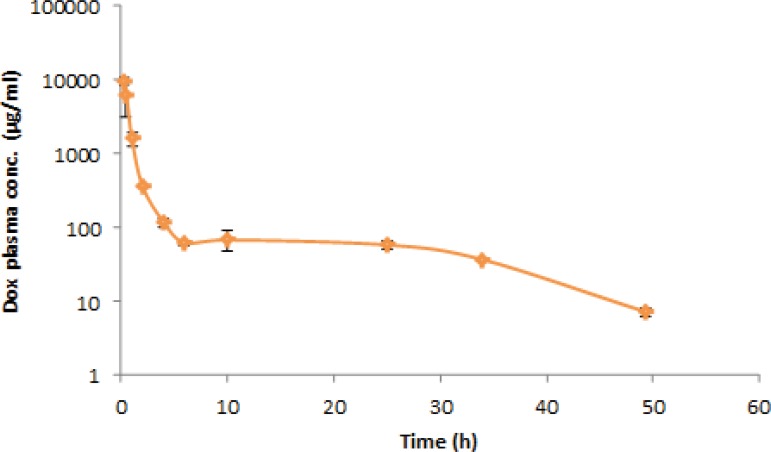
Plasma concentration–time profile after I.V. administration of doxorubicin (5 mg/Kg) in rats (n=6).

Mean plasma concentration–time profile after a single-dose administration of 5 mg/Kg of doxorubicin to six rats has been presented in the Figure 2. The visual inspection of the plasma level profile of doxorubicin shows a triexponential curve.

The pharmacokinetic parameters (mean ± SD, n=6) have been listed in the Table 5. Results are consistent with previous studies for I.V. administration of doxorubicin solution (41). As shown in the figure, the plasma level of doxorubicin could be detected until 48 h after I.V. administration. Therefore, considering the biological half life of the drug and sensitivity of the method, the drug concentration can be easily monitored for 6–7 half-lives to achieve accurate pharmacokinetic parameters. Consequently, the obtained results reveal that the proposed HPLC method is suitable for pharmacokinetic studies of doxorubicin in rats.

## Conclusion

In conclusion, a rapid and sensitive HPLC method with fluorescence detection for determining doxorubicin in plasma was reported. The advantages of the method include requiring a very small volume of plasma, simple one-step liquid-liquid extraction using minimal amount of organic solvent and excellent performance in terms of recovery and matrix effect. A simple sample preparation and short run time allow high sample throughput for pharmacokinetic studies of doxorubicin in single small species.
